# Fifty-Third Annual Meeting of the Mississippi Valley Association of Dental Surgeons

**Published:** 1896-10

**Authors:** 


### Fifty-Third Annual Meeting of the Mississippi Valley Association of Dental Surgeons.

At the annual meeting of the Mississippi Valley Dental Society, held in April, 1896, the President, Dr. W. V. B. Ames, of Chicago, delivered a very interesting address. This address has not been obtainable, and so has not been published. As may be seen from the following discussion, had upon the points presented, they were important and interesting. The treatment and management of pulpless tooth-canals called out an interesting discussion. The subject of porcelain inlays came in for free discussion.—[Ed.]

DISCUSSION OF PRESIDENT AMES' ADDRESS.

Dr. M. H. Fletcher: Mechanically, we do not always succeed in closing the foramen at the apex of the root. There is sufficient space at that point to allow septicism with all its train of *sequelæ.* My opinion is that there being more room at the apex—the foramen being larger than the tubuli—we will find more spores and germs there than can be found in the tubuli themselves, consequently inflammation, suppuration, etc., recur in this event. Such a condition may exist for years, laying dormant, until some exciting cause, as a "cold," for instance, starts the whole machine into action. I have advocated the use of arsenious acid to prevent this action. The only preparation necessary for the use of this substance is either an aqueous or alcoholic solution. It is sparingly soluble in both, though I think the latter makes the better sterilizer of the two. No matter how crooked or irregular the root canal may be, the solution may be pumped to the apex, and it is comparatively easy to dry the root canal afterward, more especially when the alcoholic solution has been used. I am more than pleased with it.

A Member: How do you prepare the solution?

Dr. Fletcher: My method is to mix equal parts of arsenious acid and prepared chalk or precipitated chalk, place this mixture in a convenient bottle and cover with pure alcohol. The latter takes up a portion of the arsenious acid, thus giving what might be called a saturated solution, though the percentage is very small. The method of introduction into the root canals is the same as that used in conveying gutta-percha solution or any of the many agents used for the purpose, *i. e.*, pumping with a suitably-sized smooth broach.

Dr. C. M. Wright: It strikes me that the method just described by Dr. Fletcher is Simon-pure theory, and before we can adopt it, it should be verified by clinical experience. Do we *know* that arsenious acid *is* a germicide? Are we satisfied by laboratory experience that it is? Of this we must also be assured before we can assume its adoption as a permanent method of treatment. Arsenious acid has been used for more than sixty years, both for devitalizing pulp tissue and obtuuding the sensibility of dentine. Following its use for the latter purpose, a crop of abscesses was the almost invariable result. If Dr. Fletcher were as minutely careful in the use of some other agent or material he would achieve an equal degree of success. I repeat, Dr. Fletcher's method is but theory as yet, though he may be working in the right direction.

Dr. H. A. Smith: The remarks just made by Dr. Wright remind me of a statement made some years ago by an eastern chemist, Chas. Mayr, to the effect that he found fungi growing upon the surface of an arsenical solution in his laboratory.

Dr. Miller is authority for the statement that the Germans have as yet no true sterilizing agent. It seems to me that the use of arsenic, as described by Dr. Fletcher, should be approached with very great caution.

Prof. Taft will not use arsenic for any purpose in the mouth, while, presumably, intelligent dentists use it freely.

Dr. Ames: Why this seeming incongruity? There are many processes brought before our conventions that are voted "no good" at the time, while, on the other hand, clinical experience demonstrates their feasibility and value.

Dr. N. S. Hoff: The president's address covers so much territory, in fact the entire field, that it could, in fact, be used instead of the official program.

Dr. Fletcher's method of treatment may be and undoubtedly is good in many cases and conditions, but it does not follow that it is so in *all*.

Arsenious acid has been used for many years to preserve cadavers for dissecting purposes, and is in use now for the same purpose. An interesting case presented itself to me not a great while ago. The patient had been taking internally for some time a nostrum containing arsenic. I found many of the teeth badly broken down, some of them mere roots; upon investigation, I discovered that the pulps in these, though dead, were not decomposed, but well preserved, mummified, in fact, or embalmed, if you please. Alcohol will dissolve less than one percent of arsenious acid. For my part, I greatly prefer to use formaline, which, properly diluted, is not escharotic.

Dr. Wright: Are we to understand that it (arsenic) will embalm living tissue?

(No answer).

Dr. H. A. Smith: With reference to the inlays mentioned in this address, I consider them in some respects superior and preferable to large masses of gold. Thermal conductivity is lessened, and that feature alone is a great comfort to the patient. The one great objection to the system of inlays is the narrow line of cement between the inlay and tooth border. In the main, the system is good and the introduction easy.

Dr. Sage: Dr. Miller states that iodoform is practically inert, notwithstanding which, many eminent practitioners firmly adhere to its use.

Prof. Taft: Dr. Fletcher may obtain good results in the exhibition of his method of treatment with arsenious acid. But I apprehend that, in the hands of many others, very undesirable results will be reached. If every case had been equally as well treated with something else, would not your success be equally as satisfactory? I used arsenious acid for a long period, mostly for obtunding sensitive dentine, but it should be used with extreme caution, if at all. I found that the pulp dies, liquifies, abscess following, and so abandoned it long ago. I do not like the principle of sealing up in a root an acrid, corrosive, dangerous agent, and see no reason for resuming its use. The young men should be cautioned against it.

Dr. Fletcher: The caution to be observed in this method, I presumed was understood. Had I been addressing a class of students, I should have expressed caution in specific terms. (Laughter).

Dr. F. A. Hunter: Do you force the solution through the foramen?

Dr. Fletcher: Not if I can help it. I have done so, with results of arsenical poisoning, abscess, etc. I have no means of *proving* my success with it, save that the proportion is larger.

Prof. Taft: It may be, that knowing the dangerous agent you were using, you observed more than ordinary care in handling it.

Dr. Fletcher: That is very likely. With proper care, such as every practitioner should always exercise, there is no great liability of accident.

Subject passed.

